# Mechanical Investigation on Fiber-Doped Cementitious Materials

**DOI:** 10.3390/polym14091663

**Published:** 2022-04-20

**Authors:** Yongcheng Ji, Yunfei Zou, Xucheng Wan, Wei Li

**Affiliations:** School of Civil Engineering, Northeast Forestry University, Harbin 150040, China; yongchengji@126.com (Y.J.); zyf13981147047@163.com (Y.Z.); w2576567000@163.com (X.W.)

**Keywords:** fiber cement-based material, mechanical investigation, volume fraction, finite element model

## Abstract

Cementitious materials can be reinforced by adding different fibers. However, the effect of different fiber reinforcements on the mechanical properties of cement-based materials remains to be further studied. This paper studies the influencing factors of different fiber cement-based materials by combining experimental and theoretical methods. The tests used carbon fiber, glass fiber, and polyvinyl alcohol (PVA) fiber-reinforced cement-based materials. The addition ratios of fibers are 0%, 0.5%, and 1% by volume respectively. The compressive strength, bending strength, and drying shrinkage are studied for 3 to 28 d. The relationship between bending strength, compressive strength, dosage, and shrinkage is analyzed. The test results show that carbon fiber cement-based materials’ bending, and compressive strength increase the fastest, followed by glass and PVA fibers. The presented mathematical model accurately predicted the strength of the three fiber cement-based materials at different curing times. Compared to glass fiber and PVA fiber, carbon fiber shrinks less. It can be shown that the fiber significantly affects the early strength change of the fiber cement-based material by changing the shrinkage size of the fiber-cement-based material. The bending strength of carbon fiber, glass fiber, and PVA fiber increases with the increase of fiber volume fraction. On the other hand, the compressive strength increases and then decreases. Mechanical tests show that carbon fiber has the best reinforcement effect. The number of fibers, center spacing, and ultimate tensile length are all important factors that affect the strength of different fiber cement-based materials. Moreover, applied ABAQUS software established compression and bending finite element models of fiber-cement composites. It can predict the mechanical performance concerning fiber cement-based materials’ different types and volume fractions.

## 1. Introduction

Cementitious materials prepared from commonly used ordinary Portland cement have several disadvantages, including lack of toughness, susceptibility to cracking, poor tensile properties, and brittleness. Bridges, expansion joints, seismic structures, blast-resistant buildings, and reinforcement patching projects all involve using cementitious materials that must be extremely strong and able to withstand tensile and bending loads. Many road and bridge constructions suffer structural damage due to cementitious material cracking, resulting in the loss of the original capacity of the roads and bridges.

**Figure 1:**
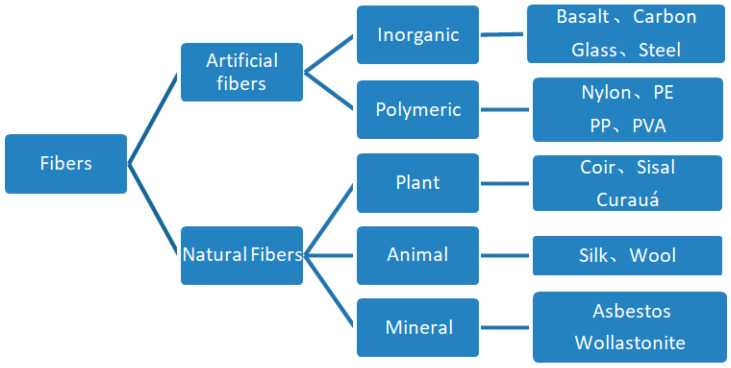
Common fibers classification.

Engineered fiber reinforced cementitious composite (ECC) is a composite material. ECC augments the capabilities by incorporating fibers, which are used to address the shortcomings of conventional cementitious materials. Figure 1 shows that the fibers commonly used to reinforce cement-based materials are mainly divided into artificial and natural fibers. Artificial fibers can be divided into inorganic and organic fibers, and natural fibers can be divided into animal, plant, and mineral fibers. Some of their features are shown in Table 1 [1,2,3,4]. The effects of various fiber compositions and lengths on cement-based materials are investigated. The addition of plant fibers to glass fiber cementitious materials alters their bending performance [5,6]. Although carbon fiber cementitious materials have greater compressive, bending, and impact strengths than pure cement, an excessive number of fibers impairs the development of mechanical properties [7,8,9]. Utilizing appropriate polypropylene fibers can improve the permeability and strength of cement mortars [10]. Fibers improve the post-peak behavior of the cement-fiber matrix from brittle to ductile and significantly increase the compressive, tensile, and bending strengths of cement materials [11,12]. PVA fibers affect cement’s strength, setting time, and morphology by increasing fracture resistance and decreasing compressive strength and setting time [13]. The addition of PVA fibers improves the specimens’ resistance to dry shrinkage and temperature shrinkage [14]. The mechanical performance of cementitious materials is strengthened by incorporating the additive of metal and polypropylene fibers [15]. At elevated temperatures, the aramid polymers retain their effectiveness within the cementitious base [16]. These are studies on improving various mechanical properties of cement-based materials by fibers. The differences in early strength and strength of various fiber cement-based materials need to be further compared and studied.

Fiber interfacial strength, fiber volume, fiber orientation, fiber length, and specimen size have distinct effects on cement characteristics [17]. The fiber-cement slurry’s interfacial area affects the strength of cementitious materials, and different curing processes modify the fiber-cement interface [18]. PVA and PP fiber cementitious composites are strong, lightweight, and deformable [19]. PVA fibers exhibit high adhesion to cement, whereas PP fibers exhibit a high degree of bridging capability [20,21]. The composite reinforcing properties of cellulose pulp and sulfate cellulose pulp significantly strengthen PVA fiber cementitious composites [22]. Loh et al. [23,24] investigated fiber hybridization, conducted mechanical tests and microstructure analysis, and determined the optimal hybridization combination using a 75% PVA/25% basalt fiber combination. Mana Halvaei et al. [25] demonstrate that when the carbon and short-cut carbon fibers are blended, crack and fracture loads and bending toughness increase dramatically with increasing fiber volume content. Numerous researchers have conducted additional research on the effect of fiber modification on the cementitious foundation. The above studies have proved the influence of various properties of fibers on cement mortar through experimental results. However, there is no theoretical proof for various factors affecting fiber cement-based materials’ strength.

Fibers have a significant enhancement effect on the mechanical properties of cementitious materials. However, there are many types of fibers on the market, and the physical and mechanical properties of different fibers are also different. This paper studies the shrinkage and mechanical properties of carbon fiber, glass fiber, and PVA fiber at different curing times. Three kinds of fiber strength prediction models with curing time are established, which provide the basis for the early strength of fiber-cement composites. In this paper, different fibers with different volume fractions are compared, and the compressive strength and bending strength are studied as a function of volume fraction to find the best fiber cement-based composites by comparison. In addition, the relationship between the bending strength and the number of fibers per unit volume, the average center distance between fibers, and the fiber pull-out length are studied in this paper, and the different effects of fiber characteristics are considered. Finally, this study uses the ABAQUS finite element method to monitor the damage process during operation and analyze the load-displacement curve. The experimental results provide technical support for the development of high-performance cementitious materials.

## 2. Experimental Program

### 2.1. Material Properties

In this study, fiber-cement composites were primarily composed of cement, fibers, fine aggregates, additives, and water. The Swan brand PO. 42.5 grade cement was used, and the cement parameters are listed in Table 2. The fine aggregate was river sand sieved to a particle size of less than 2 mm, as shown in Table 3. The water was filtered city water. The additive incorporated a polycarboxylate water reducer to ensure the fluidity of the fiber cement-based material.

**Figure 2:**
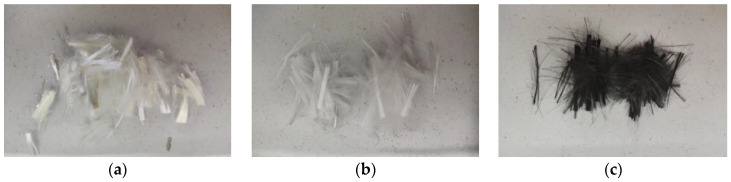
Test fibers. (**a**) PVA fiber, (**b**) Glass fiber, (**c**) Carbon fiber.

There were three types of fibers available: Carbon fiber, glass fiber, and PVA fiber, all of which have a length of 10 mm. The appearance states of various fibers are depicted in Figure 2. Carbon and glass fibers were supplied by Tianjin Carbon Company in Tianjin, China, while Shanghai Kaiyuan Chemical Technology (Shanghai, China) supplied PVA fiber. Table 4 shows the detailed material properties of the fiber material. Fiber was prepared by dry spraying and wet spinning process (the original solution was extruded from the spinneret, passed in inert gas, entered into the coagulation bath, washed off the solvent, dried, and finally stretched at high temperature to determine the radius). Length dimensions were cut as needed. The fibers were uniformly dispersed by manual tearing and then evenly sprinkled when the mortar is stirred.

### 2.2. Test Method and Mixing Ratio

#### 2.2.1. Ratio of Mixing

The mechanical and shrinkage resistance properties of cement-based composites with different fibers were investigated experimentally. A water-cement-sand ratio of 0.5:1:2 was used as the reference group. Carbon fiber, glass fiber, and PVA fiber with different volume fractions were added. According to the specification ‘Cement Mortar Strength Test Method (ISO) (GB/T 17671-2020), three representative curing times of 3 d, 7 d, and 28 d were set. The amount of water-reducing agent increased with fiber content to ensure that the specimens had higher fluidity during preparation. Table 5 shows the proportions of each mixture for all types of mixtures tested, which included an amount of nine test specimens for each mixing ratio. Three specimens were cured for 3 d, and the other six were cured for 7 d and 28 d, respectively.

#### 2.2.2. Specimen Preparation and Curing

According to the standard “Cement Mortar Strength Test Method (ISO) (GB/T 17671-2020)” and fiber properties, a total of eight steps were finally determined to ensure that the fibers were fully dispersed in Cementitious Materials.

(1) the water reducer was mixed with water evenly and added with the cement to the pot, (2) the pot was put on the holder, the machine was started, and it mixed at low speed for the 30 s, (3) sand was added evenly at the beginning of the second 30 s, (4) the fibers were evenly sprinkled and rotated at high speed for the 60 s, (5) we ensured that the fibers were sufficiently dispersed, they were rotated at high speed for the 90 s, (6) a homogeneous fiber cement mortar was poured into the oiled mold and vibrate for 20 s, (7) it was covered with a plastic wrap and cured at 20 ± 1 °C for 22 h to release, (8) the cement specimen was in the curing room for 28 d, and the curing environment was 20 ± 1 °C and 95% relative humidity.

### 2.3. Experiment Method

#### 2.3.1. Shrinkage Test

**Figure 3:**
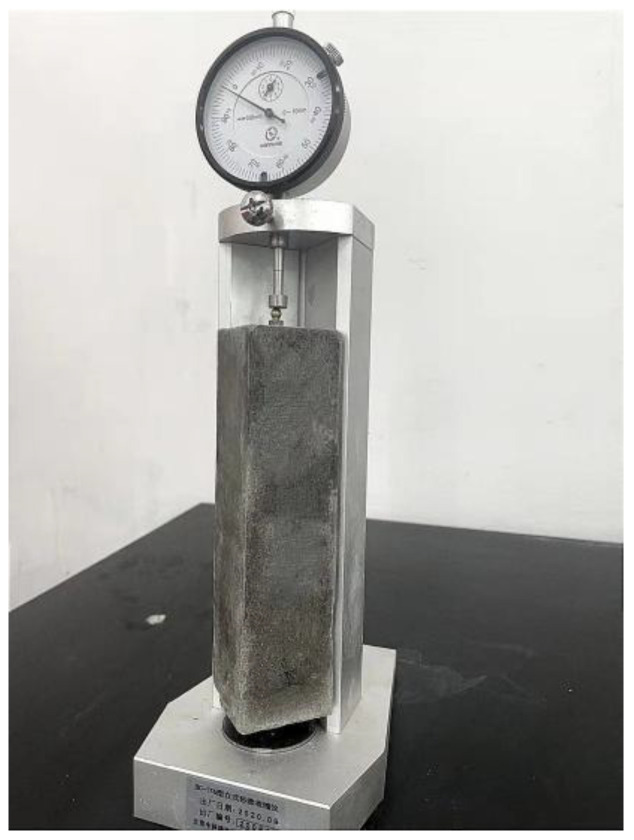
Shrinkage test.

According to the specification “Standard for test method of performance on building mortar” (JGJT70-2009), the shrinkage test adopted BC-176 vertical mortar shrinkage tester (Zhongyu Instrument, Cangzhou, China) in Figure 3. The size of the tested specimen was 40 (height) × 40 (width) × 160 mm (length) prism, and a hole with a diameter of 6.5 × 5 mm in length was opened on the two end faces of the test die. The shrinking head of the shrink instrument was fixed in the hole. After 22 h of curing time, the initial length L_0_ of the test piece was tested with a shrinkage tester. The specimens were placed in a curing box, and their shrinkage lengths were measured after 1 d, 3 d, 7 d, and 28 d, respectively. The obtained data results are the average of three specimens under the same conditions. The shrinkage value was calculated according to the following formula:

(1) $$\varepsilon_{t}=\frac{L_{0}-L_{t}}{L-L_{d}}$$

where L is the length of the specimen, L_0_ is the initial length of the test after demolding (mm), L_t_ is the measured length (mm) of the specimen at the corresponding days (1 d, 3 d, 7 d, 14 d, 28 d), and L_d_ is the upper and lower shrink head buried in the length and length of the admiral, *ε*_t_ is the shrinkage value of the corresponding days (1 d, 3 d, 7 d, 14 d, 28 d).

#### 2.3.2. Compressive and Bending Tests

**Figure 4:**
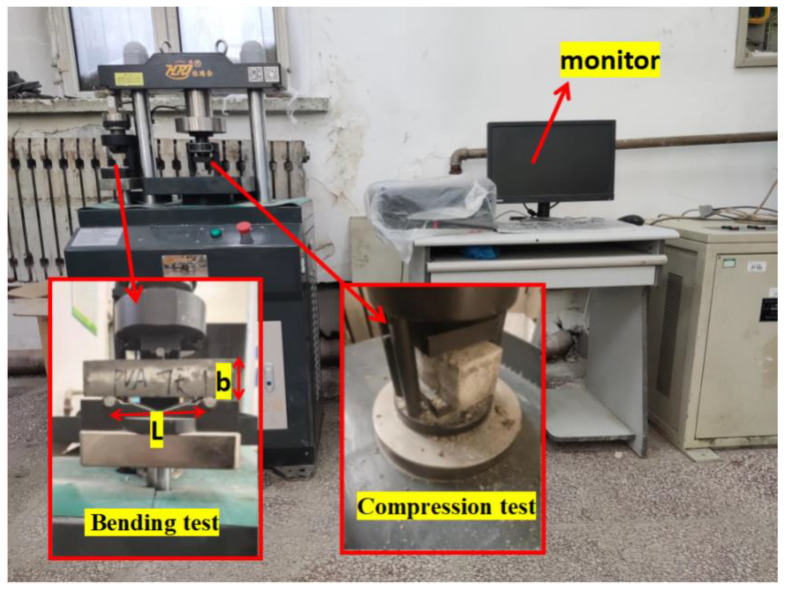
YAW-300 H testing machine.

The bending and compression tests in Figure 4 were carried out according to the “Cement Mortar Strength Test Method (ISO) (GB/T 17671-2020)”. The test is performed using a YAW-300H testing machine (Hengruijin, Jinan, China). The loading speed of the bending tester met 50 N/s ± 10 N/s. The load was evenly distributed along the width of the specimen without generating torsional stress. The size of the bending test piece was 40 (height) × 40 (width) × 160 mm (length). After the bending strength test was completed, two half-section specimens were taken out, and the compressive strength test was carried out on the side of the half-section specimens (the size of the pressure surface of the test piece was 40 mm long × 40 mm wide). In the compressive test, the load was uniformly distributed at a load speed of 2000 N/s ± 200 N/s until failure. The obtained data results are the average of three specimens under the same conditions. Error bars were added to the data analysis to indicate the degree of dispersion of the data. The bending and compressive strengths are calculated as follows:

(2) $$R_{f}=\frac{1.5F_{f}L}{b^{3}}$$

where F_f_ is the load applied when the specimen is broken (N), L is the distance between the support points of the specimen (mm), b is the side length of the prismatic section (mm), and R_f_ is the bending strength of the specimen (MPa).

(3) $$R_{c}=\frac{F_{c}}{A}$$

where R_c_ is the maximum load (N) when the specimen fails, A is the area of the compressed part of the specimen (mm^2^).

## 3. Experimental Results and Discussion

### 3.1. Strength Changes with Curing Time

**Figure 5:**
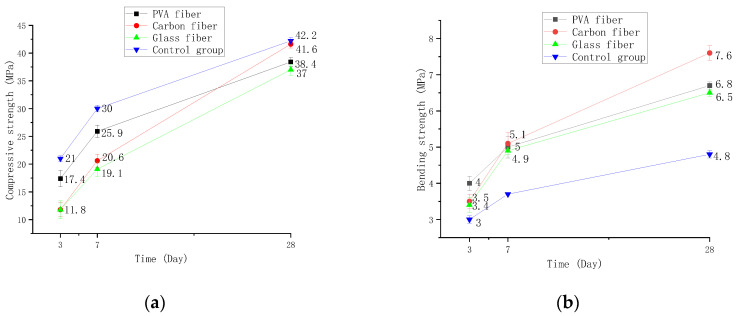
Strength changes with curing time. (**a**) Compressive strength changes, (**b**) Bending strength changes.

The effects of different fibers and curing times on the compressive and bending strength of the specimens for a certain volume admixture of 1% are shown in Figure 5. The compressive strength of control, PVA, carbon, and glass fiber cementitious materials increased by 42.8%, 48%, 61.8%, and 74% at 3–7 d, and 42.6%, 48.26%, 101%, and 93.71% at 7–28 d, respectively. The bending strength increased by 23.3%, 20%, 45.7%, and 26.4% at 3–7 d and by 29.7%, 34%, 49%, and 32.6% at 7–28 d, respectively.

The test results are analyzed as follows. The bending test at 1% volume content shows that the fiber cement-based material is significantly higher than the fiber-free cement-based material. When the fiber cement-based composite material is subjected to external stress, several cracks will be generated inside it. However, the fibers at both ends of the crack will not break immediately due to their high tensile strength, which imposes continuous stress on the crack against the crack so that the fiber can increase the bending strength of the cement-based material. PVA cement-based composites have the highest bending strength at 3–7 d, but the carbon fiber has the fastest increase rate, as high as 45.7%. The carbon fiber cement-based material is significantly higher than the other two fibers at 7–28 d, and the growth rate is the fastest, as high as 49%. The compression test at 1% volume content shows that the compressive strength of fiber doped at any stage of cement-based hardening is lower than that of doped fiber cement-based materials, and glass fiber has the most noticeable impact on its compressive strength. The strength growth rate of glass fiber cement-based materials is the fastest at 3–7 d, up to 74%. The growth rate of carbon fiber cement-based materials is the fastest at 7–28 d, as high as 101%.

It can be concluded that carbon fiber has the fastest growth rate of bending and compressive strength at 3–7 d and 7–28 d, followed by glass fiber, and PVA fiber is the slowest. The reason is that the carbon fiber has a high elastic modulus and a good contact surface with concrete, which prevents the early shrinkage effect of cement-based materials and hinders the development of cracks and leads to rapid strength growth [26].

**Figure 6:**
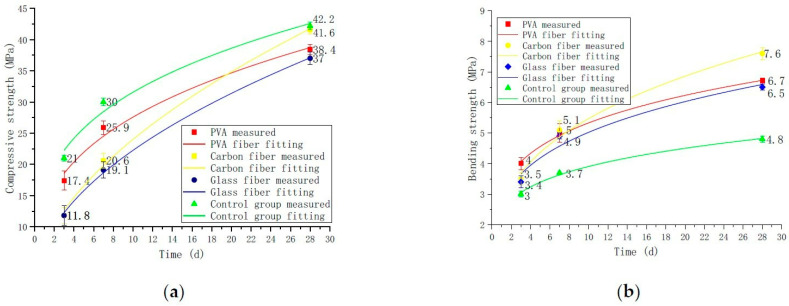
Strength changes with curing time. (**a**) Compressive strength changes, (**b**) Bending strength changes.

The conclusion of the fitting curve in Figure 6 is as follows. Because the strength of the three types of fiber cement bases varies with age in a non-linear manner, numerical simulations are used to develop strength formula models for carbon fiber, PVA fiber, and glass fiber as a function of age. It is assumed a power function, and its general form is given in Equation (4), which has two unknowns. Solving these two unknowns necessitates two separate x and y conditions. The matching intensity formula is obtained by substituting these two values into Equation (4). The fitting formulae for the compressive strength and bending strength of the three fiber cement-based materials and the cement-based materials in the control group are shown in Table 6 and Table 7. The function range has a minimum value of 0 for x = 0. The fitting curve shows that the strength proliferates in the early stages and then progressively increases in the latter stages.

(4) $$y=Ax^{B}$$

where y is the concrete strength, x is the curing time, and A and B are the undetermined fitting parameters.

Figure 6a and Table 6 show fiber cement-based materials’ constant parameters and formulas. It is found that the compressive strength of carbon fiber is the largest among the three fibers and the corresponding value of larger A and minor B. Figure 6b and Table 7 show that the relationship between the parameters and the change of bending strength and the relationship between compressive strength are consistent. The established mathematical formula model can predict the strength of the three fiber cement-based materials at different ages.

### 3.2. Change of Drying Shrinkage with Time

**Figure 7:**
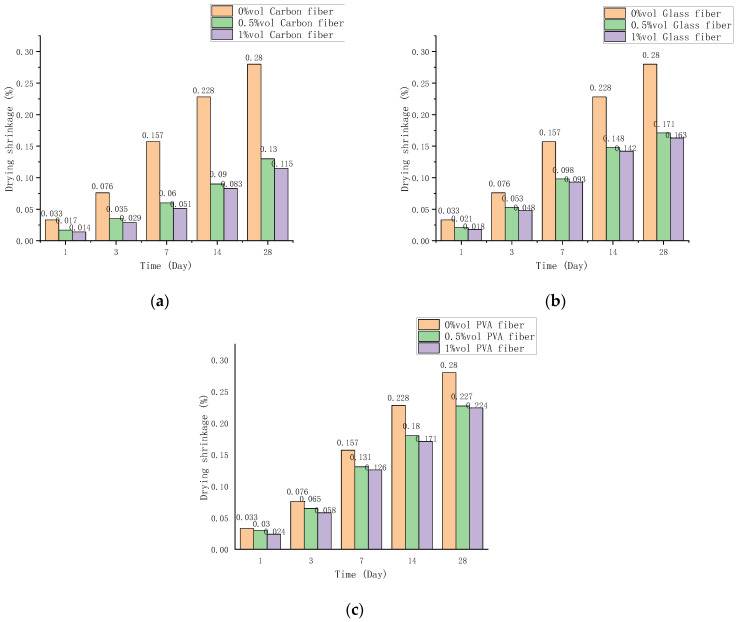
Shrinkage rate changes over time. (**a**) Carbon fiber cement base shrinkage rate change, (**b**) Glass fiber cement base shrinkage rate change, (**c**) PVA fiber cement base shrinkage rate change.

The shrinkage of fiber cement-based composites reflects the early strength. Figure 7 shows the drying shrinkage rates of three fiber cement-based materials after 1, 3, 7, 14, and 28 d. The drying shrinkage rate changes more and more with time but the rate of change gradually slows down [27]. The fiber volume content increases the drying shrinkage, but it does not change much after the volume content exceeds 0.5%. Comparing the shrinkage changes of the three fiber cement-based materials and the control group, the effect of carbon fiber is the last, followed by glass fiber and PVA fiber.

The drying shrinkage of carbon fiber cement-based composites is shown in Figure 7a. Carbon fiber may considerably decrease cement-based materials’ shrinkage response compared to the 0 content control group. On the first day, the shrinkage rate is lowered by 50%, and after 28 d, the shrinking rate will exceed 50%. The drying shrinkage variations of glass fiber and PVA fiber cement are shown in Figure 7b,c. The PVA fiber has a little inhibitory effect on cement base shrinkage, whereas the glass fiber falls somewhere in the center. The shrinkage inhibition effect of 1% by volume glass fiber reached 42% after 28 d, and 1% by volume PVA fiber reached 11%.

Correlation analysis is performed on the materials using the Pearson correlation coefficient method [28]. The correlation factors mainly include fiber type (carbon fiber, glass fiber, PVA fiber), fiber volume content (0%, 0.5%, 1%), drying shrinkage, time (3 d, 7 d, 14 d), bending strength, and compressive strength. The analyzed and processed data are shown in Table 7. ** indicates a significant correlation, and a negative sign indicates a negative correlation.

The Pearson correlation coefficients are listed in Table 8. Fiber type has a minimal link with drying shrinkage and is unrelated to age. The relationship between time and strength and drying shrinkage is found to be substantial. Compressive and bending strength have a strong relationship.

### 3.3. Change of Strength with Volume Content

#### 3.3.1. The Compressive Strength Changes with the Dosage

**Figure 8:**
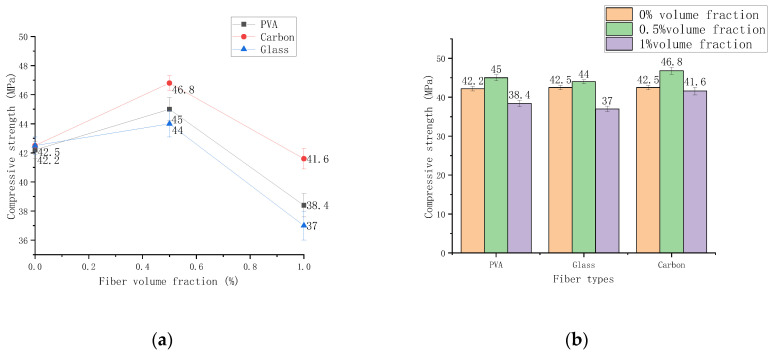
The compressive strength varies with the dosage. (**a**) Chart of compressive strength change, (**b**) Histogram of compressive strength change.

The compressive strength of PVA, glass, and carbon fibers is measured with various volume contents from 0% to 1% volume, as shown in Figure 8. The compressive strength of the three fiber cement-based composite materials at different contents increases first and then decreases with the content change, and the order is carbon fiber > PVA fiber > glass fiber.

Compared with the cement-based composite material of the control group, the strength of the three fibers with a volume content of 0.5% increased by 6.63%, 3.53%, and 10.12%, respectively. Compared with the cement-based composite material of the control group, the strength of the three fibers with a volume content of 1% decreased by 10%, 13%, and 2.2%, respectively. The test results show that the compressive strength increases slightly with the increase of fiber content but decreases when the content exceeds 0.5% volume fraction.

The analysis reasons are as follows. The first increase in compressive strength is since the fibers in the cement mortar play a specific bridging role when a small amount of uniformly distributed fibers is added to the cement-based composite material [29]. The hoop stress of the specimen during the compressive test increased, so the compressive strength of the fiber cement-based composite material mixed with 0.5% volume is improved. However, the cement-based material has many voids that are not originally mixed with excessive fiber, which will inevitably make the compressive strength of the cement-based composite material drop significantly. As a result, the compressive strength of 1% volume content is significantly reduced. Of the three types of fibers, carbon fiber has increased compressive strength compared with the other two types. This is due to carbon fiber’s strong mechanical qualities, such as elastic modulus, which prevent microcracks from developing. Increasing compressive strength is greater than the effect of reducing compressive strength generated by adding too many fibers to the test piece to enlarge the pores.

#### 3.3.2. The Bending Strength Changes with the Dosage

**Figure 9:**
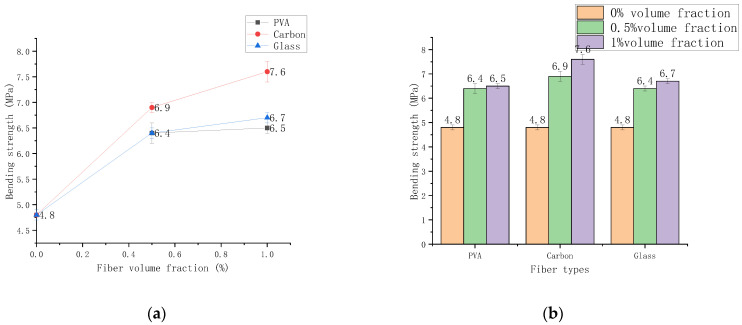
The bending strength varies with the dosage. (**a**) Chart of bending strength change, (**b**) Histogram of bending strength change.

Figure 9 shows the bending strength of PVA, glass, and carbon fibers cured for 28 d at 0%, 0.5%, and 1% volume content, respectively. The bending strength first increased rapidly, then slowly increased in which the compressive strength carbon fiber > glass fiber > PVA fiber.

The strength of the three fibers with 0.5% content and the control fiber cement-based composites increased by 33.3%, 43.7%, and 33.3%, respectively. The three fibers with 1% content had a smaller increase in strength than the 0.5% fiber-based cement-based composites that increased by 1.6%, 10.1%, and 4.7% respectively. The test results show that the bending strength increases with fiber content. The bending strength increases significantly within the range of 0–0.5% by volume and slowly increases within 0.5–1% by volume.

The analysis reasons are as follows. First, fiber may considerably lower the shrinkage rate of cement mortar, and the dry shrinkage rate of cement mortar decreases as the number of fibers rises. Second, fiber can help to prevent early plastic cracking. Compared to typical specimens, when the concentration is 0.5%, there are only fine cracks, and the load-bearing ability after cracking is also increased. Third, the tensile strength of the fiber itself plays a vital role in the influence of fiber cement-based composites. Carbon fiber has high mechanical strength, thus, incorporating it into cement-based products can significantly improve their mechanical qualities. Fourth, the fiber crack resistance mechanism is the primary reinforcing mechanism. When the carbon fiber cement-based composite material is subjected to external stress, many cracks will be generated inside it. However, the carbon fiber at both ends of the crack does not break immediately due to its high tensile strength, which stresses the crack against the crack from increasing continuously. Finally, carbon fiber can help cement-based products bend more effectively.

#### 3.3.3. Influence of Content on Bending Compressive Ratio

**Figure 10:**
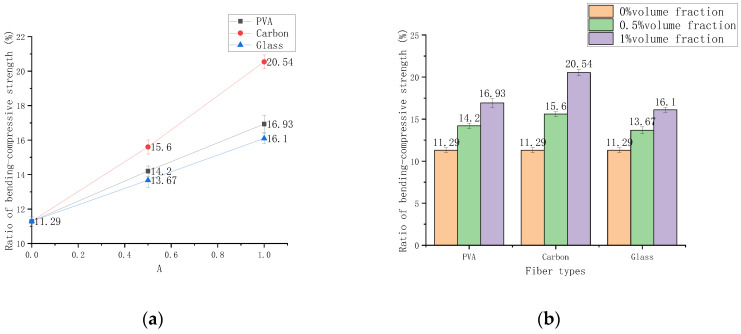
Bending compressive strength changes with volume contend. (**a**) Chart of bending compressive strength ratio, (**b**) Histogram of bending compressive strength ratio.

The bending compressive ratio can be used to reflect the crack resistance of the material to a certain extent, and it can also characterize the ductility and toughness of cement-based composites [30]. Their bending compressive ratios are tested after curing for 28 d at 0%, 0.5%, and 1% volume content of PVA, glass, and carbon fibers. The results are shown in Figure 10. Compared with the cement-based composite material of the control group, the bending compressive ratio of the three fibers with a content of 0.5% increased by 25.8%, 38.2%, and 21.1%, respectively. Compared with 0.5% fiber-added cement-based composites, the bending compressive ratios of the three fibers at 1% are increased by 19.2%, 31.6%, and 17.8%, respectively. The experimental results show that carbon fiber has the most noticeable effect on improving the crack resistance of cement-based materials. The bending compressive ratio at 0.5–1% volume content is slightly reduced compared with 0–0.5%, but the three fibers’ bending compressive ratio shows an upward trend. The use of fiber can significantly improve the toughness and ductility of the sample, but the type of fiber has little relationship with the increase in the bending compressive ratio.

### 3.4. Bending Test Failure Surface Microscope Observation

**Figure 11:**
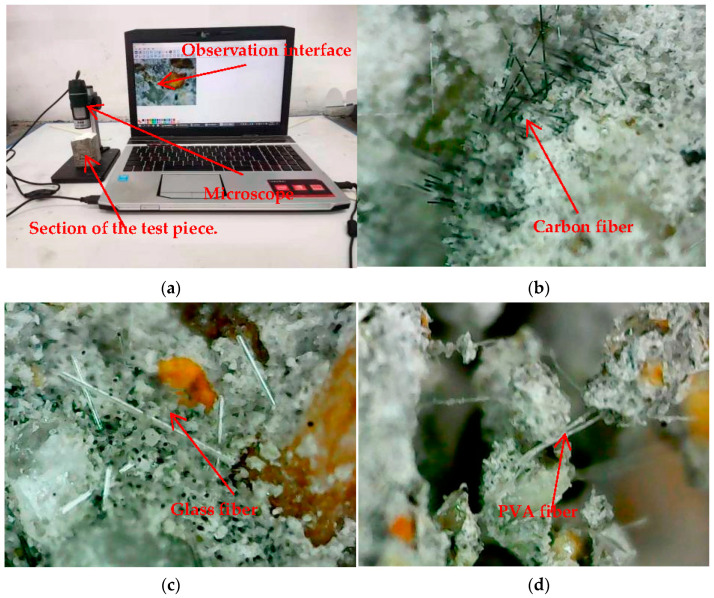
Microscope 400 times magnification for observation. (**a**) Observe the process, (**b**) Carbon fiber microscope observation, (**c**) Glass fiber microscope observation, (**d**) PVA fiber microscope observation.

The microscopic observation of the failure surface of the specimen after the bending test is shown in Figure 11a. The specimens are observed through a microscope with a magnification of 400 times. The morphology and failure morphology of three fibers in cement-based composites are observed. It can be observed that carbon fibers are densely distributed in the cement mortar in Figure 11b. The carbon fiber is black filamentous and is mainly damaged by drawing. Minor fiber slip can be clearly observed along the vertical direction of the cross-section. The further the fiber direction deviates from the vertical, the greater the length of slip that results. Glass fibers in the cement mortar can be seen in Figure 11c. The glass fibers are white filamentous and relatively scattered. It can also be observed that the fiber slip in the non-vertical direction is serious. In addition, the fractured mortar that is not detached due to fiber connection at the cross-section is also observed in Figure 11d. This observation clearly shows that the fibers have a connecting effect on the cement mortar in the bending test.

### 3.5. Bending Strength Theory Affects Calculations

It has been found that the mechanical properties of fibers are not only related to the fiber volume fraction but also to the unit volume fiber number, the average center distance between fibers, and the fiber pull-out length in cement mortar [31]. On this basis, this paper proposes three different fibers to understand the properties of fiber-cement composites better.

The volume fraction of fibers is expressed as follows:

(5) $$V_{f}=V/V_{2}=m/\rho V_{2}$$

where V is the volume of the fiber (mm^3^), V_2_ is the volume of the fiber cement-based material and 40 (height) × 40 (width) × 160 mm (length) (mm^3^), and *ρ* is the density of the fiber (kg·m^−3^), which is shown in Table 3. m represents the mass of the fiber (g).

The solution of the number of fibers (N) per unit volume of fiber cement-based composites is shown in Formula (6),

(6) $${\;N=V}_{f}{/A}_{f}L_{f}={\;V}_{f}{/(d}_{f}{/2)}^{2}{2L}_{f}$$

where A_f_ is the fiber cross-sectional area (mm^2^), and L_f_ is the fiber length (mm), d_f_ is the diameter of the fibers (mm), and these fiber parameters are presented in Table 3.

**Figure 12:**
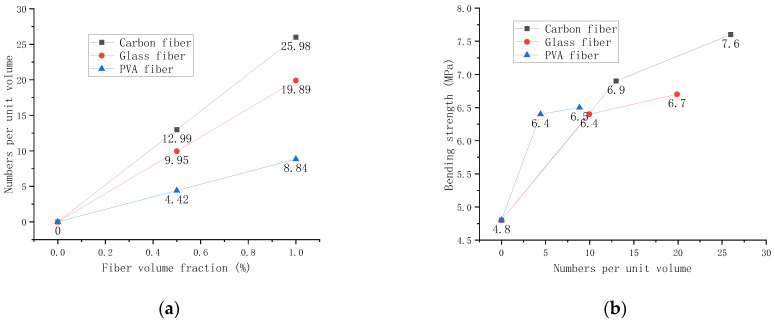
Relationship between the number of fibers and the bending strength. (**a**) Relationship between the number of fibers and the volume fraction. (**b**) Relationship between the number of fibers and the bending strength.

The Formula (6) can calculate the dosage of different fibers and different volume fractions. It can be seen from Figure 12 that the number of fibers increases with the increase of the fiber volume fraction. Carbon fibers with a minor diameter have the most significant number, followed by glass and PVA fibers. Figure 12a shows that the number of three fibers increases with the volume fraction. Among them, the number of carbon fibers is the largest, followed by glass fibers and PVA fibers. The relationship between the number of fibers and the bending strength is shown in Figure 12b. The higher the number of fibers, the higher the bending strength. However, the bending strength is not constantly increasing. The PVA reinforcement effect is the best with a small number of fibers and the same amount of fibers. However, carbon fiber has the best reinforcement effect when the number of fibers is large.

Romualdi [32] pointed out that the average center-to-center spacing of fibers is inversely proportional to crack resistance and found that when fibers are randomly distributed in the cement matrix, the average center-to-center spacing $\bar{S}$ can be calculated according to the following formula:

(7) $$\;\bar{S}=13{.8d}_{f}\;\sqrt{{1/V}_{f}}=0.6557\times \sqrt{1/m}\;$$

**Figure 13:**
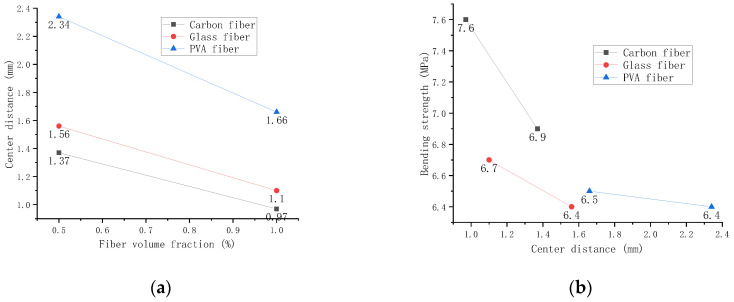
Relationship between bending strength and fiber center distance. (**a**) Relationship between volume fraction and fiber center distance. (**b**) Relationship between bending strength and fiber center distance.

Formula (7) indicates that the fiber spacing has nothing to do with length but is only related to length and fiber quality per unit volume of cement-based materials. Figure 13 shows the average among carbon fiber, glass fiber, and PVA fiber.

Figure 13a shows that fiber center distance and fiber volume fraction are inversely proportional. Among them, the center distance between PVA fibers is the largest. The relationship between the average center-to-center distance and bending strength between fibers is shown in Figure 13b, and the farther the distance, the lower the intensity. The reinforcement effect of carbon fiber is most pronounced at lower distances. However, its strength decreases more rapidly than glass fiber and PVA fiber.

Assuming that the fiber breaks at half the length, the critical pull-out length formula for carbon fiber is obtained [31]:

(8) $$\sigma_{\mathrm{fc}}^{b}=0.7246\sigma_{\mathrm{fc}}^{\mu }$$

(9) $$L_{f}^{\mathrm{crit}}{=L}_{f}\;\sqrt{\sigma_{f}^{\mu }\eta_{0}V_{f}}\;/\sqrt{2\sigma_{\mathrm{fc}}^{\mu }}\;$$

where $\sigma_{\mathrm{fc}}^{b}$ is the bending strength of the fiber cement composite in Figure 9. $\sigma_{\mathrm{fc}}^{\mu }$ is the equivalent tensile strength of fibers taking into account orientation, random distribution and bonding in mortar. $\sigma_{f}^{\mu }$ represents fiber tensile strength, and $\;\eta_{0}$ represents fiber orientation coefficient ($\eta_{0}$ = 0.2). Note: In the study of Han [31], $\sigma_{\mathrm{fc}}^{b}=2.44\sigma_{\mathrm{fc}}^{\mu }$. However, the critical pull-out length calculated in the paper and the bending strength obtained from the test can verify that the parameters given in the paper are wrong. The correct parameter should be 0.7246.

**Figure 14:**
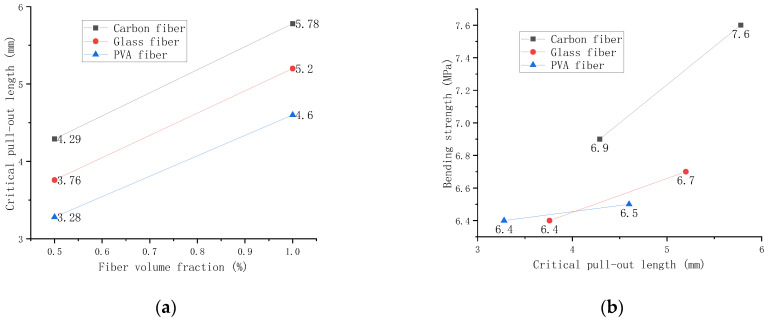
Relationship between critical pull-out length and bending strength. (**a**) Relationship between volume fraction and critical pull-out length. (**b**) Relationship between bending strength and critical pull-out length.

The indexes of various fibers in Table 2 and the bending strength of different fiber contents are substituted into Equations (8) and (9). The critical pull-out lengths of carbon fibers, glass fibers, and PVA fibers with different volume fractions in the bending test are shown in Figure 14.

Figure 14a shows that the critical drawing length increases with the volume fraction because the increase of fibers will affect the compactness of the mortar itself. Low compactness leads to reduced friction between fibers and mortar, and the critical drawing length of carbon fiber is the longest among them. However, Figure 14b shows that even if the carbon fiber has a sizeable critical drawing length and still maintains a high bending strength. It can be explained that the test machine is constantly doing work while the fibers are being pulled out, reducing the machine’s energy to the mortar itself.

## 4. Finite Element Simulation Analysis

In order to further analyze the action mechanism and difference of fibers with different physical and mechanical properties in cement mortar, this paper uses ABAQUS finite element software (ABAQUS6.14, Dassault SIMULIA, Providence, RI, USA) to simulate the test. First, the simulation utilizes Python to generate fiber code [33]. The principle of the Python code is first to determine the three-dimensional parameters of the specimen, such as 40 (height) × 40 (width) × 160 mm (length), and then import the fiber diameter, length, and volume content. The coordinates of the fibers appear randomly in the three-dimensional parameters of the specimen. It is imported into ABAQUS to build uniformly distributed and discrete fiber parts. Then, a concrete part is constructed, giving the C40 concrete damage plasticity parameters. In addition, the performance indicators, such as elasticity, plasticity, and density of glass fiber are also given. Finally, the contact between the fibers and the cement-based material is done in a “built-in” way. The mesh accuracy of the fiber part is four times that of the concrete mesh. Several compression and bending simulation tests are performed on the composite model, and the following results are finally obtained.

### 4.1. Comparison of Failure Stress Cloud Diagrams

**Figure 15:**
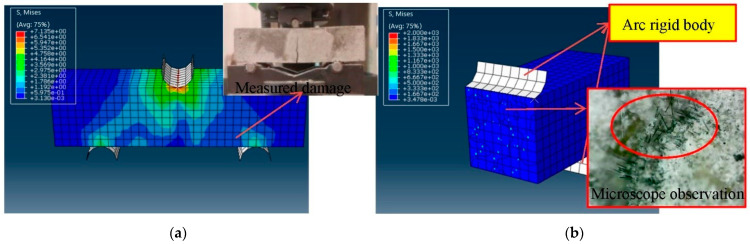
The failure process of the bending test section. (**a**) Forced destruction, (**b**) Forced destruction section.

A cloud diagram of the stress development process of the interior section is created to understand the fiber’s failure mechanism in the bending and compressive tests. Figure 15a depicts the stress distribution of the specimen in the bending test. The cement-based composite material has a stress concentration on the top during the early stages of stress, and the deformation is minimal, resulting in noticeable compression. The bottom is bent as the deformation advances, and the cement foundation is fully stretched at this point, allowing the fibers to join in the job. It can be seen that the fiber stress is much higher than that of the cement-based material, and cracks occur at the bottom. Accompanied by the expansion of the cracks, the fiber eventually exits the work, and the fiber cement base is completely destroyed. Figure 15b is the fractured section view of the bending specimen. The concentrated stress generated by the fiber tension can be observed in the section. It is observed that the fiber has a certain degree of pull-out slippage before it is broken by microscope inspection. Therefore, if the fiber is dispersed in the actual measurement and simulation, the displacement of the bending test simulation should be smaller than the value obtained from the actual measurement.

**Figure 16:**
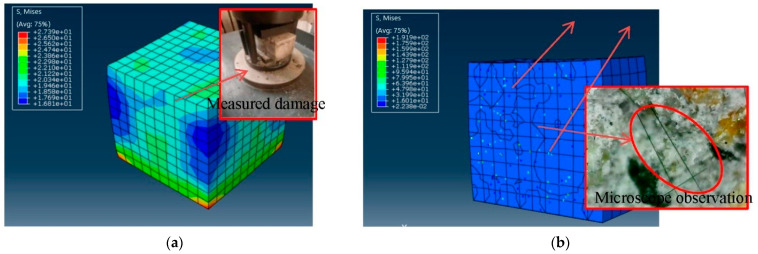
The failure process of the compression test section. (**a**) Forced destruction, (**b**) Forced destruction section.

The compression test is shown in Figure 16a,b. Under the conditions of axial compression and lateral restraint from the top and bottom, the upper and lower sides of the cement-based material are deformed and restrained, and the middle part is slightly deformed. The stress of the cement base increases from outside to inside, but it is not apparent at the initial stage. As the load increases, the deformation increases, and the strain and change of the cement-based composite material are apparent. The cracks’ development can be found from the inside to the outside, indicating that the core of the fiber cement base is damaged, and the fiber has little effect during the whole process and has almost no effect. Figure 16b is the fractured cross-section view of the compressive test piece. The fibers are randomly distributed and do not generate concentrated stress due to tension by observing the measured cross-section through a microscope. Therefore, it is further proved that the fiber has little effect on improving the compressive strength in the simulation test.

### 4.2. Comparative Analysis of Load and Displacement

**Figure 17:**
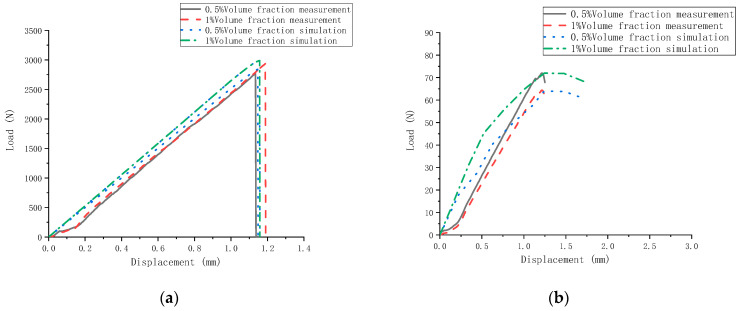

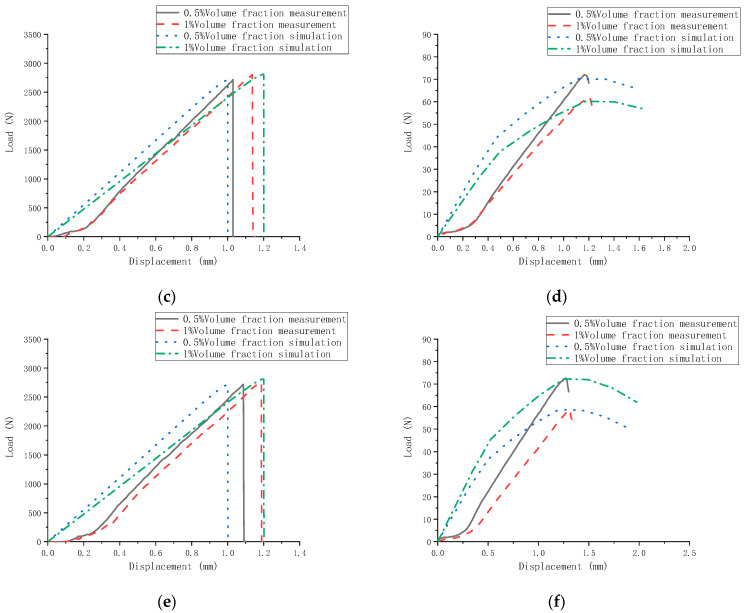
Comparison of fiber load and displacement. (**a**) Bending test comparison of carbon fiber, (**b**) Compressive test comparison of carbon fiber, (**c**) Bending test comparison of glass fiber, (**d**) Compressive test comparison of glass fiber, (**e**) Bending test comparison of PVA fiber, (**f**) Compressive test comparison of PVA fiber.

There are three types of fiber load-displacement curves. Through the load-displacement test and the simulation comparison chart, we can clearly describe the ultimate load and ultimate displacement change law of the specimen. Figure 17 compares the finite element simulation results. The load-displacement graph of the bending test rises gently before 0.2 mm. The reason is that the surface of the equipment and the cement-based composite material is not completely in contact. Comparing the fiber load displacement of 0.5% and 1% volume content, the failure displacement of the three fiber cement-based materials is between 1 mm and 1.2 mm. Whether a test or a simulation, the ultimate load and maximum displacement of 1% by volume fiber cement base are higher than 0.5% by volume, this shows that the doping of fibers can improve the toughness and ductility of the cement base and increase its bending strength. It is found that the test and the simulated failure displacement of the carbon fiber are relatively close compared to the fibers’ load-displacement relationship. The simulation test does not consider the relative slip between the fiber and the cement-based material. Therefore, it shows that the contact friction between the carbon fiber and the cement base is the largest, and its effect is the best. Both PVA and glass fiber produce relative slip, which is more consistent with the fiber observation results. PVA has the largest damage displacement in the bending test of the three fiber cement bases, followed by glass fiber and carbon fiber. The results show that the type and amount of fiber significantly affect the bending test.

Figure 17 compares the finite element simulation results, the load-displacement diagram of the compression test before 0.25 mm, and the bending behavior appears the same. The reason is that it is in the compaction stage. The test’s load displacement is tight to a straight line comparing tests and simulations, while the simulated load displacement is curved due to the selected concrete damage plastic properties. However, the change law is more consistent, and the ultimate load and failure displacement are close. Comparing the fiber load-displacement with 0.5% and 1% volume content, the failure displacements of the three fibers are all around 1.2 mm, and the simulation and experiment show consistent laws. Compared with 0.5%, the failure displacement of fiber cement-based materials with 1% volume content is slightly increased. The 0.5% volumetric fiber cement base has little effect than the 1% failure displacement, comparing the three kinds of fibers in the load-displacement relationship of the bending test. The above observations show that increasing the fiber content does not affect its toughness and strength under the compressive test, but the ultimate load is greatly reduced. The primary analysis is that the excessive fiber causes the internal cracks to develop too fast, and the damage is early, which leads to the decrease of the ultimate load.

## 5. Conclusions

The bending test, compression test, dry shrinkage test, and ABAQUS finite element simulation of carbon fiber, glass fiber, and PVA fiber with varying volume fractions and curing times are performed in this article, and the results are combined with theoretical analysis. As a result, conclusions can be drawn in the following manner:

(1) The compressive and bending strength of carbon fiber cement-based composites at 28 d increased to 41.6 MPa and 7.6 MPa, respectively, higher than those of glass fiber and PVA fiber.
(2) The higher the fiber volume content, the greater the drying shrinkage. However, the drying shrinkage remains relatively constant once the volume content exceeds 0.5%. Compared with the control group, the shrinkage resistance of 1% volume fraction carbon fiber is the best, with a shrinkage rate of 0.12% at 28 d, followed by glass fiber and PVA fiber. The strength and shrinkage of different curing days are analyzed by correlation, proving that the shrinkage is related to the early strength.
(3) A certain amount of fiber can significantly enhance the mechanical properties of cement-based materials. The bending strength of fiber cement composites increased rapidly initially and then slowly. Carbon fiber is the most significant, increasing to 6.9 MPa and 7.6 MPa at 0.5% and 1% content. The compressive strength showed a trend of first increasing and then decreasing with the content change. Carbon fiber is the most significant, increasing to 46.8 MPa at 0.5% content and decreasing to 41.6 MPa at 1% content.
(4) Theoretical calculations for the number of fibers, center spacing, and critical tensile length are performed, and their relationship to strength is analyzed. When the calculated number of fibers per mm^3^ is less than 10, the enhancement effect of PVA is most apparent. The most significant carbon fiber reinforcement effect is when the amount of fiber exceeds 10. The smaller the distance between fiber centers, the greater the strength. When the carbon fiber center spacing is 1 mm, the maximum bending strength is 7.6 MPa. Critical tensile length is inversely proportional to bending strength.
(5) Compression and bending finite element models of carbon fiber, glass fiber, and PVA fiber cement-based materials are established by ABAQUS. The finite element model can predict the mechanical properties of fiber cement-based materials with different volume fractions when comparing the load-displacement relationship between numerical simulation and experimental measurement.

## Figures and Tables

**Table 1:** Characterization of common fibers.

Material Type	Diameter (μm)	Length (mm)	Density (g/cm^3^)	Tensile Strength (MPa)	Elastic Modulus (GPa)	Elongation (%)
Carbon	6.8–20	3–18	1.57–1.80	523–4660	33–268	0.8–2.4
Glass	6–20	3–6	2.6	2000–4000	70–80	2.0–3.5
PVA	39	8–12	1.3	1600	42.8	6
PBO	13	6	1.54–1.56	5800	180–270	2.5–3.5
Steel	150–1000	13–25	7.8	350–2000	210	2–4
PE	24–38	12	0.97	1950–3000	39–100	3.1–8.0
Basalt	15–16	12	2.6–2.8	2230–4840	85.8–89.0	2.58–3.15
Aramid	12	6	1.39	3400	74	4.5
PP	12–41	6–12	0.91–0.97	850–928	2.7–9.0	7.3–30
Nylon	8	1	1.14	966	6	18
Nitinol	500–1000	/	6.45	895	41	38
Curauá	/	/	1.37–1.47	495.9 + 2.33	35.2 + 1.88	/

**Table 2:** Performance Index of Swan 42.5 Portland Cement.

Fineness Modulus	Water Requirement of Normal Consistency (%)	Stabilities	Setting Time (min)		Bending Strength (MPa)		Compressive Strength (MPa)	
			Initial Setting	Final Setting	3 d	28 d	3 d	28 d
3.2	25.4	18	160	210	5.6	9.4	25.8	45.2

**Table 3:** Particle size gradation.

Square Hole Sieve Diameter (mm)	2.00	1.60	1.00	0.50	0.16	0.08
Cumulative sieve (%)	0	7 ± 5	33 ± 5	67 ± 5	87 ± 5	99 ± 5

**Table 4:** Fiber performance indicators.

Material Type	Tensile Strength (MPa)	Elongation (%)	Fiber Density (g/cm^3^)	Elastic Modulus (GPa)	Diameter (mm)
Carbon fiber	3500	1.5	1.6	230	0.007
Glass fiber	2500	3.6	4.8	70	0.008
PVA fiber	1900	8	1.3	35	0.012

**Table 5:** The proportions of each mixture (by Weight kg/m^3^).

Mixture ID	Cement	Sand	Water	Carbon Fiber	Glass Fiber	PVA Fiber	Superplasticizer
0%	700	1400	350	0	0	0	0
Carbon 0.5%	700	1400	350	0.8	0	0	5
Carbon 1%	700	1400	350	1.6	0	0	15
Glass 0.5%	700	1400	350	0	2.4	0	5
Glass 1%	700	1400	350	0	4.8	0	15
PVA 0.5%	700	1400	350	0	0	0.65	5
PVA 1%	700	1400	350	0	0	1.3	15

**Table 6:** The compressive strength formula of different fibers.

Material Type	A1	B1	Compressive Strength Formula	R^2^
Carbon fiber	12.9	0.33	y = 12.9x^0.33^	0.985
Glass fiber	6.9	0.53	y = 6.9x^0.53^	0.994
PVA fiber	7.0	0.49	y = 7.0x^0.49^	0.997
Control group	16.4	0.29	y = 16.4x^0.29^	0.964

**Table 7:** Bending strength formula of different fibers.

Material Type	A2	B2	Bending Strength Formula	R^2^
Carbon fiber	3.17	0.22	y = 3.17x^0.22^	0.994
Glass fiber	2.57	0.32	y = 2.57x^0.32^	0.978
PVA fiber	2.73	0.26	y = 2.73x^0.26^	0.924
Control group	2.43	0.2	y = 2.43x^0.2^	0.99

**Table 8:** Pearson coefficient table.

Pearson Correlation					
	Fiber Type	Curing Day	Bending Strength	Compressive Strength	Drying Shrinkage
Fiber type	1				
Curing day	0.000	1			
bending strength	−0.354	0.832 **	1		
Compressive strength	0.210	0.920 **	0.739 **	1	
Drying shrinkage	0.536	0.785 **	0.453	0.831 **	1

** At the 0.01 level (two-tailed), the correlation is significant.

## Data Availability

All data generated or analysed during this study are included in this published article.
